# Optimal Duration of Neoadjuvant Taxane and Carboplatin Combined With Anti-HER2 Targeted Therapy for HER2-Positive Breast Cancer

**DOI:** 10.3389/fonc.2021.686591

**Published:** 2021-06-08

**Authors:** Yifan Xie, Siyu Wu, Ying Zhang, Jianwei Li, Miao Mo, Zhimin Shao, Guangyu Liu

**Affiliations:** ^1^ Department of Breast Surgery, Fudan University Shanghai Cancer Center, Shanghai, China; ^2^ Department of Oncology, Shanghai Medical College, Fudan University, Shanghai, China; ^3^ Department of Cancer Prevention & Clinical Statistics Center, Fudan University Shanghai Cancer Center, Shanghai, China

**Keywords:** breast cancer, human epithelial growth factor receptor 2, neoadjuvant chemotherapy, pathologic complete response, event free survival

## Abstract

**Background:**

Taxane, carboplatin and trastuzumab (TCH) is an effective neoadjuvant regimen for human epidermal growth factor receptor 2 (HER2)-positive breast cancer with high pathologic complete response (pCR) rate. The KATHERINE trial changes the outlook for high-risk HER2-positive breast cancer, which suggests that escalation treatment for patients with residual disease after neoadjuvant anti-HER2 therapy may improve survival. The major objective of this study was to investigate the fewest cycles of neoadjuvant TCH therapy needed to screen out non-pCR patients.

**Methods:**

This retrospective study included patients with HER2-positive breast cancer who received either four or six cycles of TCH preoperatively at Fudan University Shanghai Cancer Center between 2008 and 2019. The pCR status was evaluated, and relevant factors associated with pCR were identified using univariate and multivariable analyses. The pathological results of core needle biopsy (CNB) in the breast tumor after two cycles of neoadjuvant chemotherapy were also collected. Kaplan-Meier curve was used to estimate the event-free survival (EFS).

**Results:**

Of 758 eligible patients, 303 were included and analyzed in the four-cycle group and 455 in the six-cycle group. There was no significant difference between the two groups in terms of the pCR rate (46.5% [95% CI 40.9% - 52.2%] in the four-cycle group and 49.9% [95% CI 45.3% - 54.5%] in the six-cycle group, p = 0.365) or the four-year EFS (90.8% in four-cycle group and 93.8% in six-cycle group; p = 0.264). Multivariable analysis indicated that a negative hormone receptor status and the weekly paclitaxel were independent factors for predicting pCR. After adjusting for factors in the multivariable analysis, there was still no significant difference between four and six cycles of neoadjuvant TCH (OR = 1.252, 95% CI 0.904 - 1.733, p = 0.176). Furthermore, 17.9% patients with invasive carcinoma on CNB after two cycles of TCH ultimately achieved pCR in the breast after the completion of neoadjuvant treatment.

**Conclusion:**

Four cycles of taxane/carboplatin-based neoadjuvant anti-HER2 therapy may be applied as an optimal treatment duration for screening high-risk HER2-positive breast cancer patients for escalation treatment. Further prospective study is warranted.

## Introduction

Neoadjuvant therapy was originally administered with the aim of reducing the size of primary tumors. Several clinical studies (1–3) have shown that early response to initial cycles of neoadjuvant chemotherapy is correlated with long-term survival. Therefore, pathologic complete response (pCR) has been widely adopted as a surrogate marker for long-term efficacy: patients who achieve pCR in both the breast and axilla after neoadjuvant therapy have a better prognosis, leading to improved disease-free and overall survival, especially in human epidermal growth factor receptor 2 (HER2)-positive breast cancer.

In contrast, residual tumors are usually an independent poor prognostic sign. Patients with residual disease after neoadjuvant chemotherapy with HER2-directed therapy are at a high risk of relapse, leading to worse survival outcomes than patients with no residual disease (1–3). Therefore, this subset of high-risk patients has a high unmet medical need for effective therapy in the early disease stage. The KATHERINE trial (4) changes the outlook for high-risk HER2-positive breast cancer, which suggests that escalation treatment for patients with residual disease who have been screened out *via* neoadjuvant chemotherapy is a must. There is no doubt that the risk of invasive disease recurrence or death can be reduced by 50% among HER2-positive breast cancer patients with residual tumors who switch from HER2-directed therapy to the single agent T-DM1 (4). The trial provides a brand-new remedy for HER2-positive patients at high risk and brings us into the practice-changing KATHERINE era. In such circumstances, the purpose of applying the neoadjuvant approach has been extended from initial disease downstaging, thereby permitting breast conservation to test the sensitivity of neoadjuvant therapy. This can subsequently provide prognostic information to allow escalation or de-escalation in the adjuvant setting.

In the presence of anti-HER2 blockade, taxane-carboplatin-based chemotherapy has shown similar efficacy to that of anthracycline or anthracycline-taxane, with less toxicity (5, 6). A series of clinical trials and cohort studies (4, 7–10) also revealed a satisfactory pCR rate with the combination of taxane (T), carboplatin (C) and trastuzumab (H) in the neoadjuvant setting.

While taxane-carboplatin based chemotherapy has been used for various cycles in different trials (4, 5, 9, 10) and in practice, the optimal duration of taxane, carboplatin and anti-HER2 agent as a neoadjuvant regimen remains unclear. Thus, in this large retrospective study, we first investigated the optimal cycles of neoadjuvant TCH therapy on pCR rate and survival.

## Methods

### Patients

**Figure 1:**
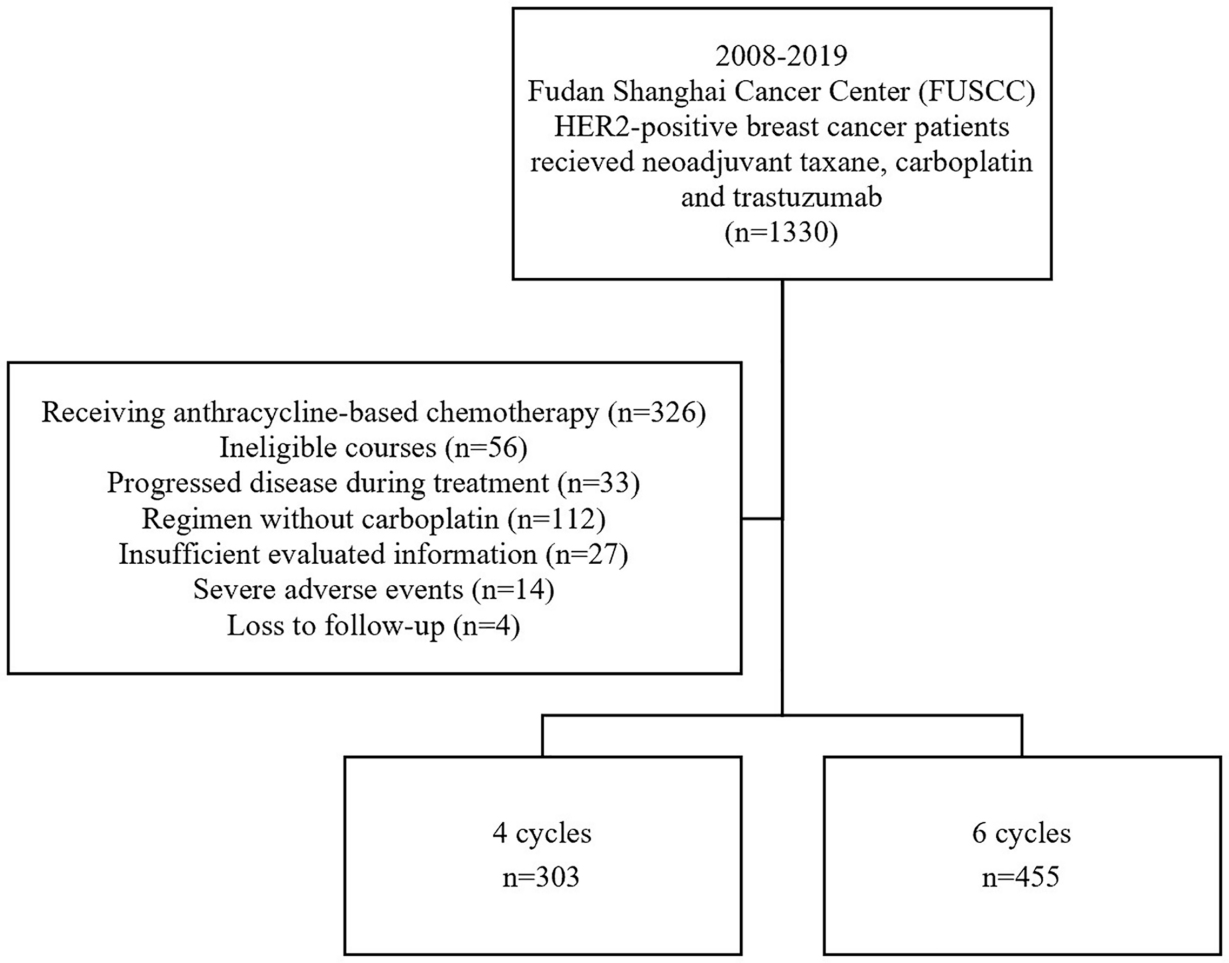
Flow chart of patient selection.

We reviewed all the medical records of patients who received four or six cycles of TCH preoperatively at Fudan University Shanghai Cancer Center (FUSCC, Shanghai, China) between 2008 and 2019. Detailed inclusion criteria included (1) untreated HER2-positive breast cancer with histologically confirmed tumor, (2) age between 18-70 years; no inflammatory breast cancer; (3) assessable tumor in the breast without evidence of distant metastasis, (4) Eastern Cooperative Oncology Group performance status ≤1, (5) left ventricular ejection fraction > 55%, (6) no history of other cancer except *in situ* uterine cervix cancer or skin basal cell carcinoma; (7) all patients received either four or six cycles of paclitaxel/docetaxel and carboplatin combined with trastuzumab before lumpectomy or mastectomy. The exclusion criteria ruled out (1) patients who received anthracycline-based chemotherapy or a regimen without carboplatin before surgery, (2) patients who received more than six cycles or fewer than four cycles of TCH for any reason, (3) patients with insufficient evaluated information, (4) with severe adverse events (including cardiac adverse events, grade 4 hematologic toxicity and grade 3/4 gastrointestinal toxicity) for treatment discontinuation, (5) patients who could not be analyzed due to loss of follow-up. The complete flow chart is shown in **Figure 1**. The protocol was reviewed and approved by the independent ethical committee/institutional review board, and all patients included have given their written informed consent.

### Treatment

All eligible patients were treated according to a schedule comprising taxane, carboplatin and trastuzumab every three weeks or every week for four or six courses. Treatment schedule and courses were formulated by multidisciplinary team based on the local guidelines, which recommended four cycles and every-three-week schedule before 2013. For the every-three-week schedule, patients received paclitaxel 175 mg/m^2^ or docetaxel 75 mg/m^2^, carboplatin at an AUC of 6 and trastuzumab 6 mg/kg (loading dose 8 mg/kg) on day 1 of a three-week cycle. For the weekly schedule, patients received paclitaxel 80 mg/m^2^ and carboplatin at an AUC of 2 on day 1, 8, 15 of a four-week cycle, with trastuzumab 2 mg/kg (loading dose 4 mg/kg) every week. The duration of neoadjuvant treatment was 12 or 16 weeks in the four-cycle group and 18 or 24 weeks in the six-cycle group. Response evaluations were performed every two treatment cycles, and additional core needle biopsy (CNB) was recommended since 2015 when patients had completed the initial two cycles of chemotherapy to determine the presence of residual malignant tumor tissue. This ultrasound-guided CNB was performed using a 14-gauge cutting needle at the site of the treated breast within the baseline skin-tattooed area. At least three tissue specimens were required from different sections of the lesion in order to decrease the false-negative rate. After neoadjuvant treatment, proper surgeries were performed followed by up to one year of trastuzumab. Radiotherapy was recommended based on clinical characteristics and pathologic results according to the local institute guideline. Hormone receptor (HR)-positive patients received endocrine therapy for at least five years. Anthracycline-based regimen was widely used for patients with residual tumor in the four-cycle group as adjuvant therapy. However, postoperative adjuvant chemotherapy was not mandatory and varied in the rest of the patients.

### Assessment

Clinical staging was determined using the 8^th^ edition of the American Joint Committee on Cancer (AJCC) staging manual. The estrogen receptor (ER), progesterone receptor (PR), Ki-67 and HER2 status were determined using immunohistochemistry (IHC). In this study, HR-negative was defined as both ER and PR < 1% and HR-positive was defined as ER or PR ≥ 1%. HER2-positive was determined by HER2 3+ on IHC or a positive result on fluorescence *in situ* hybridization (FISH). The endpoint of this study was pCR rate. pCR was defined as no invasive carcinoma in both breast and axilla. Event-free survival (EFS) was calculated as the interval from the first date of neoadjuvant treatment to the earliest occurrence of disease progression, locoregional recurrence, distant metastases, or death from any cause. The follow-up duration of patients without any event was counted to the date of last follow-up.

### Statistical Analysis

Patient and tumor characteristics are expressed as percentages or median values. Continuous variables were compared by the Mann-Whitney test, while categorical variables were compared by Pearson’s chi-squared test or Fisher’s exact test. Binary logistic regression was used in the multivariable analysis. Survival is presented by the Kaplan-Meier curve and was assessed with the log-rank test. Multivariable analysis for survival was evaluated by Cox model. A p value < 0.05 (two-sided) was considered significant. All statistical analyses were performed using SPSS v.20.0 (SPSS Company, Chicago, IL, USA).

## Results

### Patient Characteristics

A total of 758 patients were included in this large, observational, retrospective study at the FUSCC (**Figure 1**); 303 (40.0%) patients received four courses of treatment, and 455 (60.0%) received six courses of treatment. The median age was 50 years. Fifty-seven percent of patients (n = 435) were premenopausal and forty-three percent (n = 323) were postmenopausal. Regarding stage, 258 (34.0%) patients had stage I or stage II disease, 493 (65%) patients had stage III disease and 7 patients could not be categorized due to insufficient records. The HER2 status of all patients was positive, with IHC 3+ or a positive FISH result with IHC 2+. In total, 364 patients (48.0%) were HR+/HER2+ and 394 (52.0%) were HR-/HER2+. There were 124 (16.5%) patients receiving triweekly paclitaxel, 47 (6.3%) receiving triweekly docetaxel and 579 (76.4%) patients receiving weekly paclitaxel in neoadjuvant treatment (p = 0.003). There were no significant differences between the four-cycle and six-cycle groups in terms of patient or tumor characteristics (**Table 1**). However, patients in four-cycle group (30.7%, n = 93; 95% CI 25.5% - 35.9%) were more likely to receive anthracycline-based adjuvant treatment than six-cycle group (7.3%, n = 22; 95% CI 2.0% - 5.5%, p < 0.001). And the six-cycle group (18.0%, n = 82; 95% CI 4.3% - 10.2%) had higher conservation rate than the four-cycle group (7.3%, n = 22; 95% CI 14.5% - 21.6%, p < 0.001).

**Table 1:** Clinicopathologic characteristics of study population.

	Four cycles (n=303)	Six cycles (n=455)	p value
Median age	49.3	49.8	0.483
Median tumor size (mm)	43.2	42.6	0.797
Menopausal status		
Premenopausal	168 (55.4)	267 (58.7)	0.378
Postmenopausal	135 (44.6)	188 (41.3)	
Clinical tumor stage			
T0-2	173 (57.1)	242 (53.2)	0.290
T3-4	130 (42.9)	213 (46.8)	
Clinical nodal stage			
N0	53 (17.7)	91 (20.2)	0.396
N1-3	246 (82.3)	359 (79.8)	
NA	4	5	
Clinical stage			
I-II	107 (35.6)	151 (33.6)	0.573
III	194 (64.4)	299 (66.5)	
NA	2	5	
Estrogen receptor status			
Positive	132 (43.6)	210 (46.2)	0.483
Negative	171 (56.4)	245 (53.8)	
Progesterone receptor status			
Positive	110 (36.3)	162 (35.7)	0.862
Negative	193 (63.7)	292 (64.3)	
NA	0	1	
Ki-67			
<20%	17 (6.3)	31 (7.1)	0.684
≥20%	254 (93.7)	408 (92.9)	
NA	27	21	
Taxane treatment			
Triweekly paclitaxel	56 (18.7)	68 (15.1)	0.003
Triweekly docetaxel	8 (2.7)	39 (8.7)	
Weekly paclitaxel	236 (78.7)	343 (76.2)	
Breast surgery			
Mastectomy	281 (92.7)	373 (82)	<0.001
Lumpectomy	22 (7.3)	82 (18.0)	
Adjuvant therapy			
Anthracycline-based	93 (30.7)	17 (3.7)	<0.001
Anthracycline-free	210 (69.3)	438 (96.3)	

### Tumor Response

**Figure 2:**
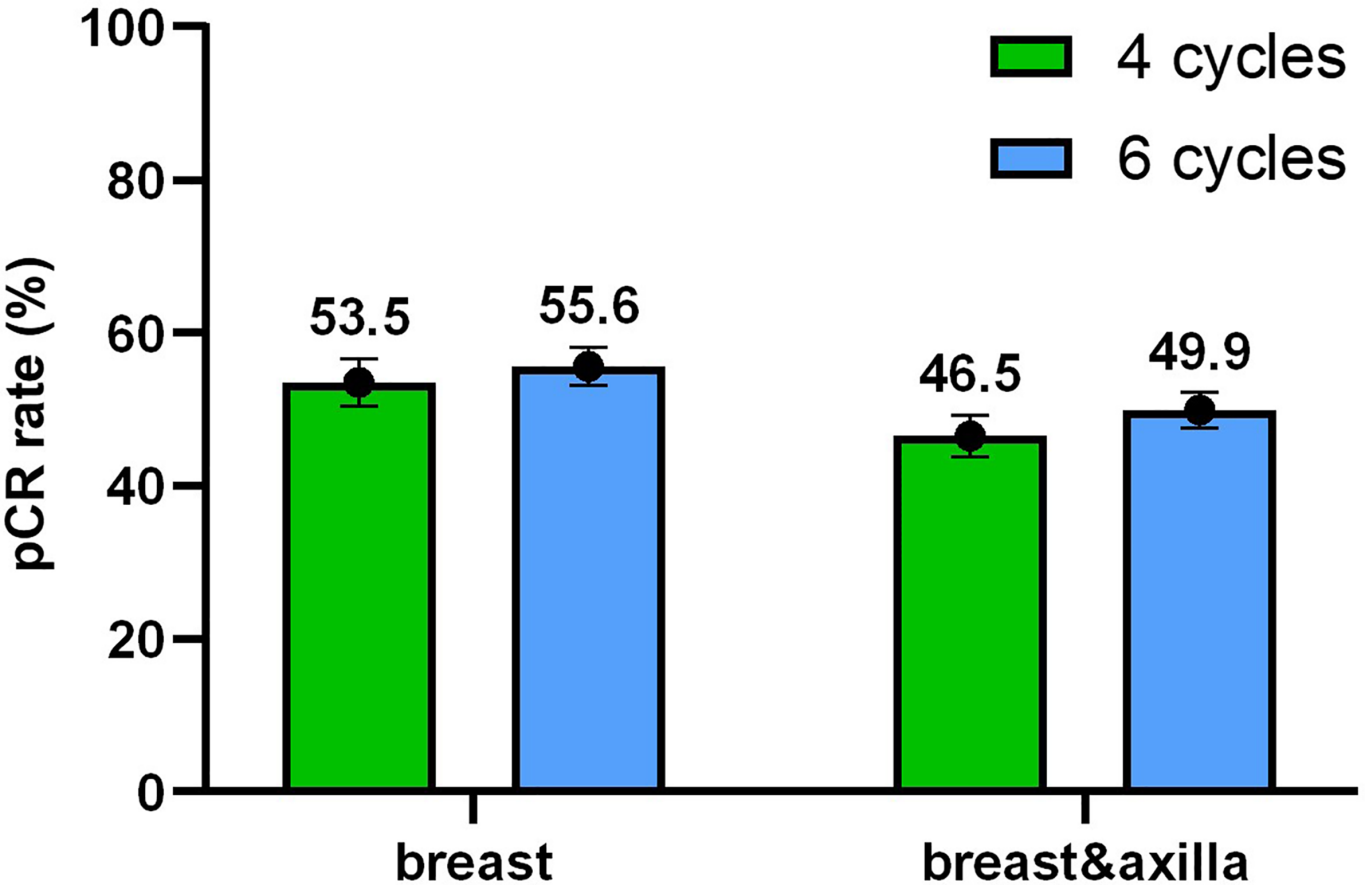
Tumor response. The pathologic complete response (pCR) rate with 95% confidence intervals in the breast and in the breast and axilla depended on different cycles.

The tumor response results of the two groups are shown in **Figure 2**. In the four-cycle group, over half of patients had no residual invasive disease in the breast and nearly three quarters of patients had no residual disease in the axilla, which indicates that 46.5% (n = 141; 95% CI 40.9% - 52.2%) of patients achieved pCR. As for six cycles, a slightly higher proportion (49.9%, n = 227; 95% CI 45.3% - 54.5%) of patients achieved pCR in both the breast and axilla without significant difference (p = 0.365).

**Figure 3:**
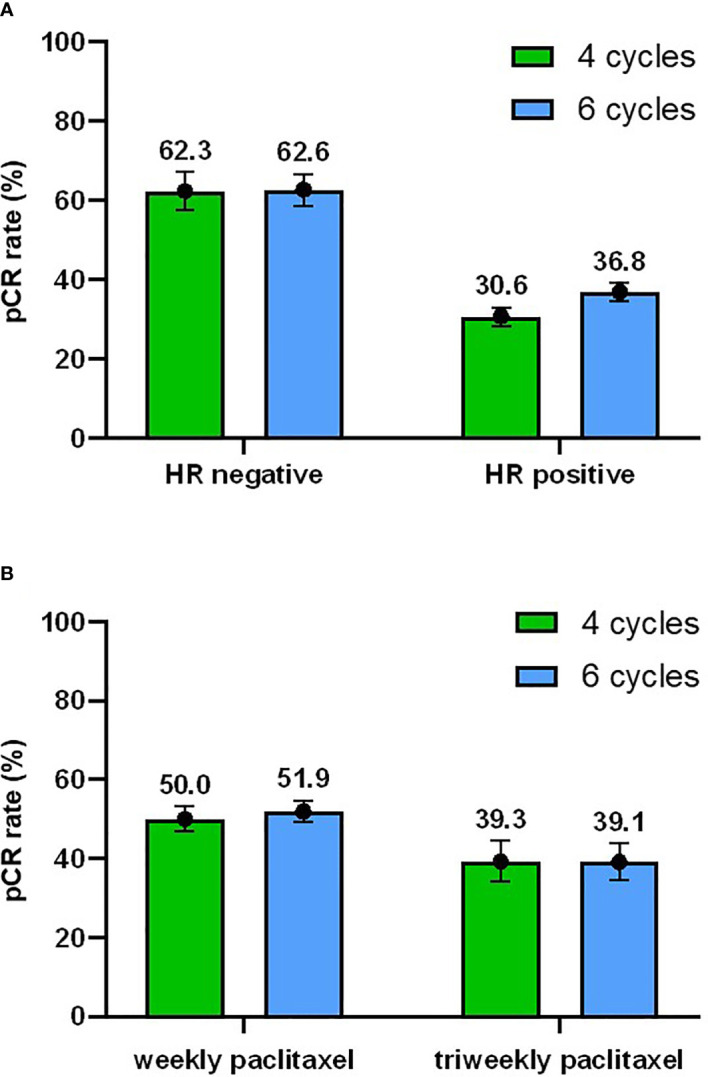
Subgroup analyses. **(A)** Subgroup analysis of pCR rate with 95% confidence interval with respect to the hormone receptor (HR) status. **(B)** Subgroup analysis of pCR rate with respect to the taxane regimens.

Factors that might influence the pCR rate were added to univariate and multivariable analyses (**Table 2**). The HR status (p < 0.001) and paclitaxel treatment (p = 0.025) were significantly associated with pCR. After adjusting for all factors (age, menopausal status, clinical tumor stage, clinical nodal stage, HR status, Ki-67, and taxane treatment) in the multivariable analysis, there were still no significant differences between four-cycle and six-cycle groups (OR = 1.252, 95% CI 0.904 - 1.733, p = 0.176, **Table 2** and **Figure 3**).

**Table 2:** Subgroup analysis of the correlation between the pCR rate and number of cycles.

	pCR (n=368)	non-pCR (n=390)	Univariate analysis p value	Multivariable analysis OR^*^ (95% CI), p value
Age				
<50	176 (47.8)	178 (45.6)	0.547	0.866 (0.563-1.333), 0.514
≥50	192 (52.2)	212 (54.4)		
Menopausal status				
Premenopausal	216 (58.7)	219 (56.2)	0.479	0.886 (0.575-1.366), 0.584
Postmenopausal	152 (41.3)	171 (43.8)		
Clinical tumor stage				
T0-2	207 (56.2)	208 (53.3)	0.270	0.806 (0.588-1.104), 0.179
T3-4	161 (43.8)	182 (46.7)		
Clinical nodal stage				
N0	75 (20.3)	70 (18.0)	0.352	0.961 (0.646-1.430), 0.843
N1-3	294 (79.7)	319 (82.0)		
NA	4	5		
Hormone receptor status				
Negative	244 (66.3)	150 (38.5)	<0.001	0.314 (0.229-0.430), <0.001
Positive	124 (33.7)	240 (61.5)		
Ki-67				
<20%	16 (4.7)	32 (8.7)	0.033	1.781 (0.918-3.456), 0.088
≥20%	326 (95.3)	336 (91.3)		
NA	27	21		
Taxane treatment				
Triweekly paclitaxel	49 (13.5)	75 (19.4)	0.079	
Triweekly docetaxel	22 (6.0)	25 (6.5)		1.521 (0.712-3.249), 0.279
Weekly paclitaxel	293 (80.5)	286 (74.1)		1.679 (1.069-2.637), 0.025
Number of cycles				
4 cycles	141 (38.3)	162 (41.5)	0.365	1.252 (0.904-1.733), 0.176
6 cycles	227 (61.7)	228 (58.5)		

*The prior one of every subgroup (age<50, premenopausal, T0-2, N-, HR-, Ki-67<20%, triweekly paclitaxel, four cycles) is set as the reference of OR.

*pCR, pathologic complete response; non-pCR, residual invasive disease was found either in the breast or axilla in the surgical specimens.

*Odds ratio (OR)>1 represents higher odds of achieving pathologic complete response.

Of note, 374 patients underwent CNB after the completion of two cycles of neoadjuvant treatment from 2015 to 2019 and 40.4% of patients (n = 151; 95% CI 35.4% - 45.4%) showed residual invasive carcinoma in the CNB specimens. However, 17.9% (27/151; 95% CI 11.7% - 24.1%) of them achieved pCR according to the final breast surgical specimens. And in 223 patients with negative results of biopsy, 22.9% (51/223; 95% CI 17.3% - 28.4%) of them still remained residual disease after the completion of neoadjuvant treatment, including 10 cases in four-cycle group and 41 cases in six-cycle group.

### Subgroup Analysis

Based on the results of the univariate and multivariable analyses, the subgroup analyses were focused mainly on the HR status and paclitaxel treatment. In total, 62.3% (95% CI 54.6% - 69.9%) of HR-negative patients and 30.6% (95% CI 22.9% - 38.2%) of HR-positive patients achieved pCR in the four-cycle group and 62.6% (95% CI 56.3% - 68.8%) vs 36.8% (95% CI 30.4% - 43.2%) achieved pCR in the six-cycle group (**Figure 3A**). Subgroup analyses according to the HR status showed that patients with HR-negative tumors had a higher pCR rate regardless of the treatment duration. In the four-cycle group, the pCR rate was 50.0% (95% CI 43.6% - 56.4%) when the weekly paclitaxel was adopted, while it was 39.3% (95% CI 26.1% - 52.5%) when the triweekly paclitaxel was used. In the six-cycle group, the pCR rate was 51.9% (95% CI 46.6% **-** 57.2%) vs 39.1% (95% CI 27.3% - 50.9%) for the weekly and triweekly schedule, respectively (**Figure 3B**). Thus, patients who received weekly treatment also had a better tumor response than those who received triweekly paclitaxel in both the four-cycle and six-cycle groups.

### Event Free Survival Analysis

**Figure 4:**
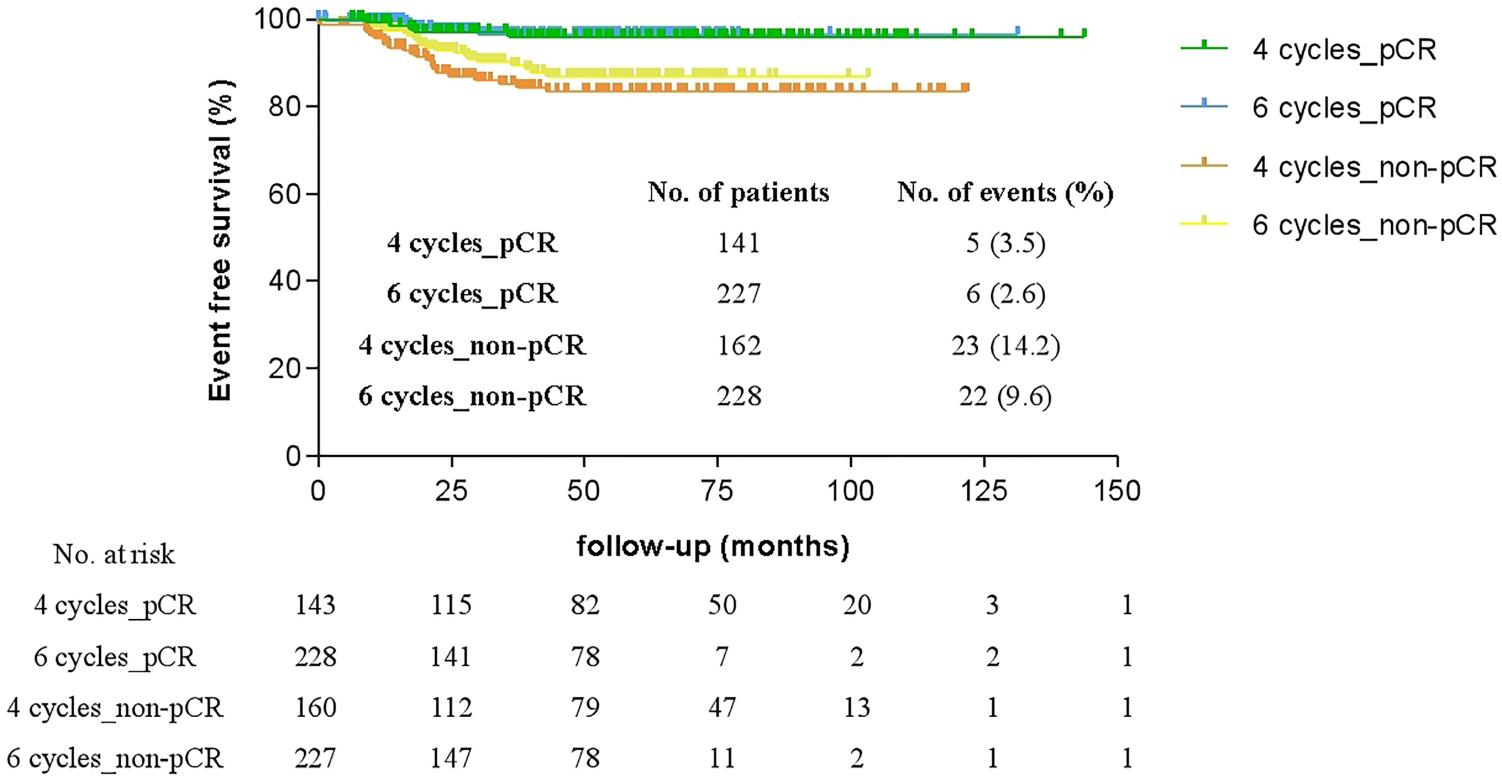
Comparison of four-year event-free survival (EFS) of patients who achieved pathologic complete response (pCR) or with residual disease (non-pCR) after four or six cycles of neoadjuvant therapy.

**Figure 4** shows the Kaplan-Meier survival analysis for the EFS of HER2-positive patients who received either four or six cycles of TCH neoadjuvant chemotherapy. The median follow-up time was 48.2 months, thus four-year EFS was calculated and assessed. There was a significant difference in the four-year EFS regardless of whether patients achieved pCR (97.0% vs 88.4%, p < 0.001). In contrast, there was no significant difference between four cycles and six cycles in either patients were pCR (96.5% vs 97.4%, p = 0.823) or patients with residual disease (85.6% vs 90.3%, p = 0.259). And four-year EFS was not influenced by any potential factors including adjuvant anthracycline-based regimens (**Supplementary Table 1**).

## Discussion

Previous studies have reported that the pCR rate of HER2-positive breast cancer patients receiving neoadjuvant therapy with taxane, carboplatin plus trastuzumab regimen ranges from 44%-64.9% (7, 9, 11, 12) for four courses and from 34.1%-57.5% (6, 8, 10, 13–21) for six courses. However, there were no existing data pertaining to a direct comparison of these two courses. Our study demonstrated similar high pCR rates of 46.5% and 49.9% for four cycles and six cycles of neoadjuvant TCH, respectively, which was consistent with previous findings. Since this was a retrospective study, to avoid nonnegligible bias, we conducted multivariable analysis. HR-negative status and weekly paclitaxel were the only two independent factors associated with a higher pCR rate. This result is consistent with those of several clinical trials, such as the TRYPHAENA study (18) and the TRAIN-2 study (5), as well as our previous study (10). A meta-analysis suggested that when combined with single anti-HER2 agent, significant improved pCR rate should be seen in paclitaxel group compared with docetaxel (22). However, our results showed no significant difference, which was not consistent with previous studies. We speculated that it might be due to the limited number of patients in the docetaxel group (n = 47, including no patients receiving weekly docetaxel) and the retrospective nature of our study. Identical reasons might cause the significant difference of taxanes treatment and surgical options. After adjusting for such factors, there was still no significant difference in the pCR rate between four and six cycles of neoadjuvant TCH. Our findings in the current study cohort suggest a similar pathological response, with a promising argument for a shorter duration of neoadjuvant therapy.

Neoadjuvant chemotherapy combined with anti-HER2 targeted therapy is now widely used to downstage disease as well as provide data on the pathological response, which can act as a surrogate for long-term efficacy. The KATHERINE trial (4) indicated that compared to continuation of trastuzumab adopted in the neoadjuvant setting, escalation of treatment to T-DM1 in the adjuvant setting can lower the risk of relapse and death among patients with residual disease by 50%. Even patients with minimal residual tumors can benefit from T-DM1. The KATHERINE trial opened a new era wherein the treatment strategy of high-risk HER2-positive breast cancer has been renewed by escalating adjuvant therapy in patients with residual invasive disease after pre-surgical treatment. Since two additional cycles of TCH may not alter the final tumor pathological response status, the decision of applying postoperative escalation therapy could be made as early as after the completion of four cycles of neoadjuvant therapy. In addition, we found that in at least 27 patients who ultimately achieved pCR after receiving additional two or four cycles of neoadjuvant TCH, residual invasive carcinoma could still be detected on CNB after two cycles of neoadjuvant treatment. It indicated us that only two cycles of neoadjuvant treatment were insufficient for a part of patients achieving pCR. Therefore, we conjectured that four cycles might be the optimal duration for neoadjuvant TCH in this aspect.

Our findings also suggest a potential de-escalating strategy by shortening the total chemotherapy courses to four cycles in those patients who achieve pCR. In this study, patients who achieved pCR had significantly better EFS regardless of the duration of neoadjuvant anti-HER2 therapy, although the postoperative adjuvant treatment varied in the group of patients who received four cycles of TCH. A comprehensive meta-analysis (23) showed that the five-year EFS of pCR patients treated with subsequent adjuvant chemotherapy was 86%, whereas that in patients who did not receive additional chemotherapy was 88% (p=0.60), which indicated that patients who achieved pCR after neoadjuvant chemotherapy would not benefit further from additional cytotoxic chemotherapy in the adjuvant setting. The regimen comprising 6 cycles of adjuvant taxane and carboplatin combined with trastuzumab was first established by the BCIRG 006 trial in the 2000s and has already been proven to benefit survival compared with the control (24). However, four cycles of weekly PH (paclitaxel and trastuzumab) and TCtxH (docetaxel, cyclophosphamide and trastuzumab) were both de-escalating anti-HER2 regimens with good long-term outcomes in early-stage breast cancer patients in the adjuvant setting (25, 26). Therefore, four cycles of systemic chemotherapy combined with one year of lower toxic targeted therapy might be sufficient in selected patients whose tumors show excellent responses to anti-HER2 therapies.

There are several potential limitations to this study. First, treatment group allocation was nonrandomized. Although we conducted multivariable analysis to adjust differences, significant potential biases may still exist. Therefore, further prospective investigations are necessary. Second, no patients receiving dual anti-HER2 agents were included in this study. As neoadjuvant pertuzumab was not approved by National Medical Products Administration (NMPA) of China until 2019, few patients received pertuzumab therapy at our center. The NeoSphere trial has already showed improved pCR rate when pertuzumab combined with TCH, which afterwards took trastuzumab and pertuzumab as standard targeted therapy for preoperative treatment. Therefore, whether our conclusion can apply to the setting of dual HER2 inhibition remained unclear and our results should be verified with a dual-targeted regimen. Lastly, patients with residual invasive disease after surgery who received four cycles of neoadjuvant chemotherapy were administered different adjuvant therapies, and the number of events is small. These may have influenced the survival results.

## Conclusions

We demonstrated that four cycles might be an optimal duration of neoadjuvant TCH therapy to determine further escalating or de-escalating therapy according to the pathological response of HER2+ breast cancer.

## Data Availability Statement

The original contributions presented in the study are included in the article/**Supplementary Material**. Further inquiries can be directed to the corresponding authors.

## Ethics Statement

All the procedures performed in studies involving human participants were in accordance with the ethical standards of the institutional and/or national research committee and with the 1964 Helsinki declaration and its later amendments or comparable ethical standards.

## Author Contributions

YX and SW participated in the conceptualization and carried out the investigation, data collection, statistical analysis and drafted the manuscript. YZ and JL participated in the investigation, data collection, and helped to draft the manuscript. MM participated in the data collection and statistical analysis. ZS and GL conceived of the study and participated in its design and coordination and helped to draft the manuscript. All authors contributed to the article and approved the submitted version.

## Conflict of Interest

The authors declare that the research was conducted in the absence of any commercial or financial relationships that could be construed as a potential conflict of interest.
