# Supplementary material for: Optimal Duration of Neoadjuvant Taxane and Carboplatin Combined With Anti-HER2 Targeted Therapy for HER2-Positive Breast Cancer
Source: Front Oncol. 2021 Jun 8;11:686591. doi: 10.3389/fonc.2021.686591 (PMC8217668; doi:10.3389/fonc.2021.686591)
Supplement: Supplementary file 1 [file Table_1.docx]

**Supplementary Table S1** Analysis of potential factors associated with four-year event-free survival.

|  | | Univariate analysis  HR^*^ (95% CI) | p | Multivariable analysis  HR^*^ (95% CI) | p | |
| --- | --- | --- | --- | --- | --- | --- |
| Age  <50  ≥50 | 0.660 (0.389-1.121) | 0.124 | 0.736 (0.334-1.622) | 0.447 | |  |
| Menopausal status  Premenopausal  Postmenopausal | 0.793 (0.462-1.362) | 0.401 | 0.941 (0.420-2.180) | 0.882 | |  |
| Clinical tumor stage  T0-2  T3-4 | 1.476 (0.589-3.699) | 0.406 | 1.197 (0.473-3.025) | 0.704 | |  |
| Clinical nodal stage |  |  |  |  | |  |
| N0  N1-3 | 1.460 (0.754-2.825) | 0.262 | 1.411 (0.704-2.827) | 0.332 | |  |
| Hormone receptor status  HR-  HR+ | 1.029 (0.610-1.738) | 0.914 | 0.754 (0.427-1.331) | 0.330 | |  |
| Ki-67  <20%  ≥20% | 3.483 (0.481-25.195) | 0.216 | 4.266 (0.586-31.037) | 0.152 | |  |
| Taxane treatment  Triweekly paclitaxel  Triweekly docetaxel  Weekly paclitaxel | 0.453 (0.059-3.494)  0.802 (0.430-1.497) | 0.448  0.489 | 0.000 (0.000-3.974e+286)  0.774 (0.408-1.468) | 0.971  0.433 | |  |
| Number of cycles  4 cycles  6 cycles | 0.742 (0.439-1.254) | 0.266 | 0.756 (0.421-1.356) | 0.348 | |  |
| Pathologic response  non-pCR  pCR | 0.257 (0.133-0.497) | ＜0.001 | 0.261 (0.130-0.523) | ＜0.001 |  |  |
| Adjuvant therapy  Anthracycline-based  Anthracycline-free | 1.723 (0.941-3.158) | 0.078 | 1.387 (0.710-2.711) | 0.339 |  |  |

*The prior one of every subgroup (age<50, premenopausal, T0-2, N-, HR-, Ki-67<20%, triweekly paclitaxel, four cycles, anthracycline-based, non-pCR) is set as the reference of HR

* pCR: pathologic complete response; non-pCR: residual invasive disease was found either in the breast or axilla in the surgical specimens
